# Crack Detection of Reinforced Concrete Structure Using Smart Skin

**DOI:** 10.3390/nano14070632

**Published:** 2024-04-05

**Authors:** Yu-Jin Jung, Sung-Hwan Jang

**Affiliations:** 1 Department of Smart City Engineering, Hanyang University ERICA, Ansan 15588, Republic of Korea; yujin0421@hanyang.ac.kr; 2 Department of Civil and Environmental Engineering, Hanyang University ERICA, Ansan 15588, Republic of Korea

**Keywords:** carbon nanotube, polyurethane, crack detection, self-sensing, structural health monitoring

## Abstract

The availability of carbon nanotube (CNT)-based polymer composites allows the development of surface-attached self-sensing crack sensors for the structural health monitoring of reinforced concrete (RC) structures. These sensors are fabricated by integrating CNTs as conductive fillers into polymer matrices such as polyurethane (PU) and can be applied by coating on RC structures before the composite hardens. The principle of crack detection is based on the electrical change characteristics of the CNT-based polymer composites when subjected to a tensile load. In this study, the electrical conductivity and electro-mechanical/environmental characterization of smart skin fabricated with various CNT concentrations were investigated. This was performed to derive the tensile strain sensitivity of the smart skin according to different CNT contents and to verify their environmental impact. The optimal CNT concentration for the crack detection sensor was determined to be 5 wt% CNT. The smart skin was applied to an RC structure to validate its effectiveness as a crack detection sensor. It successfully detected and monitored crack formation and growth in the structure. During repeated cycles of crack width variations, the smart skin also demonstrated excellent reproducibility and electrical stability in response to the progressive occurrence of cracks, thereby reinforcing the reliability of the crack detection sensor. Overall, the presented results describe the crack detection characteristics of smart skin and demonstrate its potential as a structural health monitoring (SHM) sensor.

## 1. Introduction

Reinforced concrete (RC) structures are essential to modern infrastructure, underpinning critical facilities like bridges, high-rise buildings, and dams. Despite their inherent strength and durability, these structures are susceptible to cracking due to environmental factors, load variations, and material aging. Early detection and monitoring of these cracks are vital for preserving structural integrity and ensuring safety. Traditional structural health monitoring (SHM) methods, such as visual inspections and standard sensing techniques, have proven inadequate [1,2,3,4,5]. They often miss small cracks that can lead to significant damage, are labor-intensive, and lack continuous, real-time monitoring. This shortfall in effective monitoring poses a risk, especially for structures critical to public safety and urban life. Consequently, there is a pressing need for advanced, real-time monitoring technologies in SHM for RC structures. Such innovative solutions would greatly enhance SHM’s accuracy and contribute to the longevity and safety of these essential structures, marking a significant advancement in civil engineering and ensuring the resilience and safety of our infrastructure.

In recent years, the field of SHM has experienced remarkable advancements, driven by the development of smart material sensing technologies using carbon-based nanocomposites. Carbon nanomaterials such as carbon nanotubes (CNTs), graphene, graphite carbon, and hybrid nanostructures are robustly used due to their thermal and mechanical stability, and electrical and electronic properties. Among these, CNTs are known for their unique structural feature of an elongated bundle shape, which provides higher mechanical reinforcement with polymer composites and superior flexibility in stretchable electronics compared to other carbon nanomaterials [6,7,8,9]. Additionally, their cost-effectiveness, compared to other materials, affords them a high level of selectivity as a key nanomaterial for application in large-scale structures such as civil, aviation, and space infrastructures in SHM applications [10,11,12,13]. For example, Park et al. [14] developed a glass fiber-reinforced plastic skin embedded with CNT fiber sensors to implement SHM in aircraft structures. The CNT fiber sensors showed strain measurement results similar to conventional strain gauge measurements. In addition, Nonn et al. [15] observed crack propagation behavior using CNT films for electrical impedance tomography imaging. They confirmed that cracks were clearly observed through the electrical impedance tomography program as they grew larger. Olejnik et al. [16] synthesized a composite by printing CNT onto polyurethane (PU) and investigated the potential for structural damage detection based on the increase in sensor signal with increasing deformation. Furthermore, the critical role of mechanical properties inherent in nanostructured materials should not be overlooked, as emphasized by Magazzù, A. et al. [17]. Echoing this perspective, Szeląg, M. [18] confirmed the beneficial impact of structural integration enabled by the mechanical characteristics of CNT’s nanostructure, which facilitate cohesive connections leading to enhanced bending tensile strength.

The strain sensing capability of CNT-based composites is considered a key requirement for future SHM techniques and applications. To address these challenges and requirements, several studies have attempted to develop novel self-sensing nanocomposites for such applications [19,20,21,22]. Lu et al. [23] manufactured conductive aggregates coated with CNT polymer nanocomposites and evaluated their sensitivity to deformations in concrete structures caused by external factors. They used materials coated with CNT polymer nanocomposites at a 20 wt% CNT content to compare the electrical resistivity under cyclic compressive loads. The maximum fractional change in the resistivity value under periodic compression was around 80%, indicating somewhat low detection performance. Castañeda-Saldarriaga et al. [24] developed a cement composite containing CNTs for detecting damage due to deformation and conducted characterization tests of its piezoresistive behavior. Consequently, the optimal CBM/CNT concentration was determined to be 0.8 w%. They focused on investigating the electrical properties induced by piezoresistance to demonstrate its suitability for SHM applications. Meoni et al. [25] developed cement materials integrated with CNTs to assess sensitivity to structural changes, observing an increase in electrical resistance due to the formation of compressive cracks. However, they faced issues with inconsistent gauge factors and complete loss of detection capability after initial crack formation. Additionally, there was no evaluation on how environmental conditions affect the materials’ response. The reviewed CNT-based sensors for civil structures rely solely on electrical changes that occur immediately upon crack formation, leading to somewhat inaccurate detection performance. In such instances, errors can arise from electrical changes caused by minor shocks. Furthermore, in real scenarios, it is crucial to monitor crack growth post-occurrence. Most studies discuss embedded CNT sensors, which pose significant challenges such as being difficult to repair and apply to existing concrete structures.

In this study, we propose a smart skin composed of a CNT/PU composite for detecting cracks in RC structures. The smart skin is applied as a coating on the exterior of structures to serve as a sensor monitoring the occurrence and growth of cracks. To develop the optimal sensor, the electrical conductivity at various CNT concentrations is investigated. Following this, the electrical properties under mechanical and environmental changes are analyzed to evaluate the smart skin’s tensile strain sensitivity and environmental impact. Finally, the applicability, reproducibility, and reliability of the smart skin as a crack detection sensor are assessed upon its application to structures.

## 2. Materials and Methods

### 2.1. Materials

**Figure 1:**
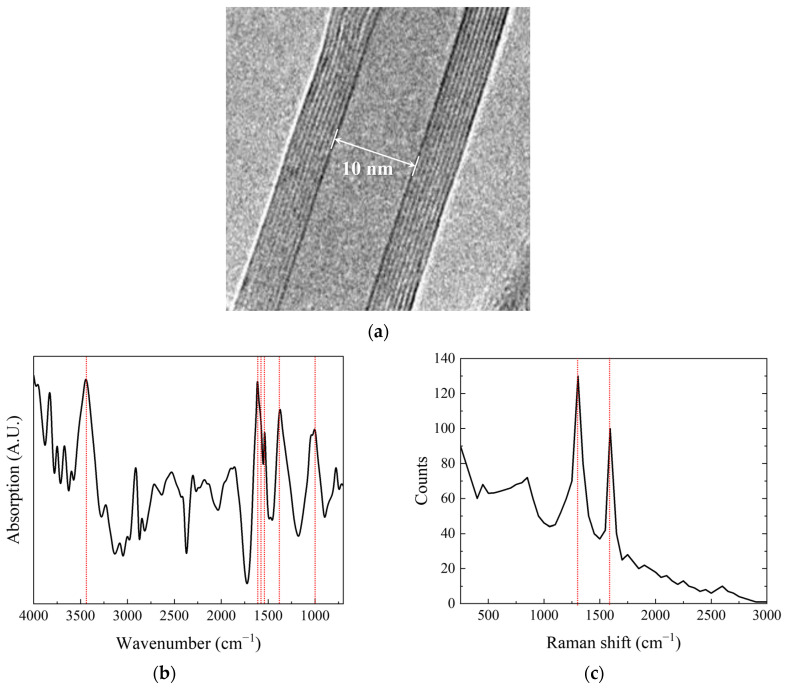
(**a**) TEM image, (**b**) FTIR spectrum (red line: peak value) and (**c**) Raman spectrum of CNTs. Reprinted with permission (red line: mode D and G) [26,27,28]. Copyright 2017, NanoLab, Inc. (MA, USA).

CNTs were acquired from Nanolab (Waltham, MA, USA), with diameters ranging from 10 to 30 nm, lengths varying nominally from 5 to 20 microns, and a carbon purity greater than 85 wt%. The impurities include iron and ceramic oxides. The characteristics of the CNTs are displayed in Figure 1. Figure 1a shows a transmission electron microscopy image of the CNTs, revealing their multi-walled nature [26]. Here, we observe a 10 nm inner diameter, 9 concentric walls, and a clear inner channel. The Fourier-transform infrared (FTIR) spectrum of the CNTs is depicted in Figure 1b [27]. Peaks appeared at 1000, 1375, 1540, 1575, 1615, and 3450 cm^−1^, which can be attributed to C=O stretching, C–O, and OH stretching of the carboxylic acid group, respectively, formed on the side wall of the CNTs. Figure 1c presents the Raman spectrum of the CNTs, featuring two characteristic bands: mode D (1304.3 cm^−1^) and mode G (1593.3 cm^−1^) [28]. In Figure 1b,c, the red lines indicate peak values in Figure 1b and modes D and G in Figure 1c, respectively. A medium-flexibility PU casting resin was purchased from Easy Composite (Stoke-on-Trent, UK), characterized by a viscosity range of 450–650 mPa·s, a curing time of 1–2 h at room temperature, and a tensile strength of 3.4–3.8 MPa. The solvent used for dispersing CNTs was high-purity acetone, with a 99.7% purity level, supplied by Samjeon Chemical (Gyeonggi, Republic of Korea).

### 2.2. Fabrication Procedure

**Figure 2:**
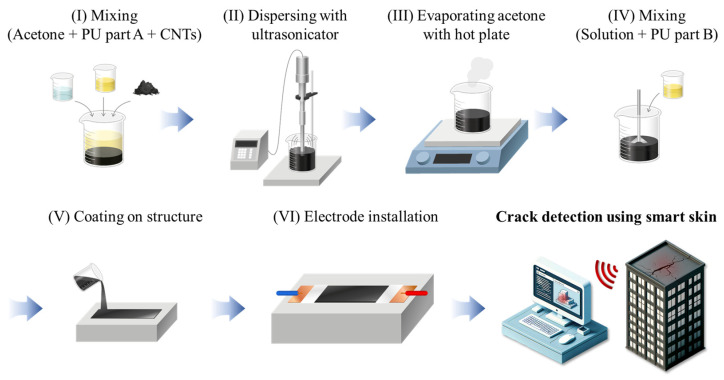
Fabrication procedure of smart skin.

The smart skin was fabricated by uniformly dispersing CNTs within a PU matrix, employing the same methodology as previous studies [29,30,31], as illustrated in Figure 2. Approximately 50 mL of acetone was used as a dispersing agent to dissolve the high-viscosity PU resin, facilitating the high-quality dispersion of CNTs. The dispersion of CNTs within the PU matrix was achieved using a Q700 sonicator (Qsonica, Newtown, CT, USA). Ultrasonic dispersion breaks the strong van der Waals forces between the CNTs, resulting in a homogeneous mixture. To minimize the overheating effects of the sonicator, the mixture-containing beaker was placed within a larger beaker filled with ice, and the sonicator was operated in pulse mode (15 s on, 15 s off) for a duration of 80 min. The total energy output was approximately 100,000 J. The dispersed mixture was then placed on a hot plate at 60 °C for 24 h to evaporate the acetone. A PU hardener was added to the mixture in the same ratio as the PU resin, and it was thoroughly mixed using a TR50M three roll mill (Trilos, San Ramon, CA, USA). The mixture was then coated to a constant thickness of 0.7 mm, and to minimize voids in the samples, it was subjected to vacuum drying for 24 h. Afterward, the hardened samples were cut into pieces measuring 70 mm × 10 mm. For the electrical conductivity measurements of the smart skin, samples were prepared with varying CNT concentrations ranging from 0 to 7 wt%.

### 2.3. Characterization

The microstructure of CNTs within the PU matrix was observed using a Mira 3 scanning electron microscope (Brno, Czech Republic) at 15 kV. The samples were coated with a thin layer of platinum using a Q300T sputter coater. The electrical conductivity of the smart skin was calculated by measuring the electrical resistance of the samples using a Keithley 2450 (Keithley, Solon, OH, USA). Samples with high electrical resistances above $10^{9}$ Ω were measured using a Keithley 6517B (Keithley, Solon, OH, USA). The electrical resistance of the samples was measured using the two-point probe method. To minimize measurement errors due to contact resistance between the probe tips and the sample, copper tape was attached to both ends of the sample, and high-purity silver ink was applied between the sample and the copper tape [32,33]. The electrical conductivity of the samples was calculated using Equation (1):

(1) $$\sigma =\frac{L}{AR}$$

where σ (S/m) is the electrical conductivity of the smart skin, R (Ω) is the electrical resistance of the smart skin, A ($m^{2}$) is the area of the electrode, and L (m) is the distance between electrodes.

For the electro-mechanical/-environmental characterization, smart skin samples with CNT concentrations of 2 wt%, 3 wt%, and 5 wt% were prepared. Electro-mechanical characterization was conducted using a servohydraulic test system equipped with a 100 kN load cell. The system applied a pure tensile force to the samples at a displacement rate of 5 mm/min. Concurrently, the electrical resistance of the samples was measured using a Keithley DMM 6500 (Keithley, Solon, OH, USA). Electro-environmental characterization was performed using a temperature and humidity chamber. The electrical resistance of the samples was measured using a Keithley 2700 (Keithley, Solon, OH, USA). Tests were conducted based on standard environmental conditions of 20 °C room temperature and 45% relative humidity, with temperature and humidity ranges adjusted between −10 °C and 70 °C and 20% and 80%, respectively.

## 3. Results and Discussion

### 3.1. Electrical Conductivity of Smart Skin

**Figure 3:**
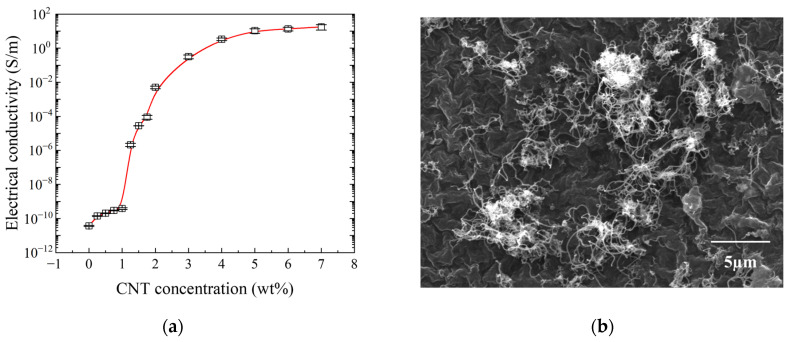

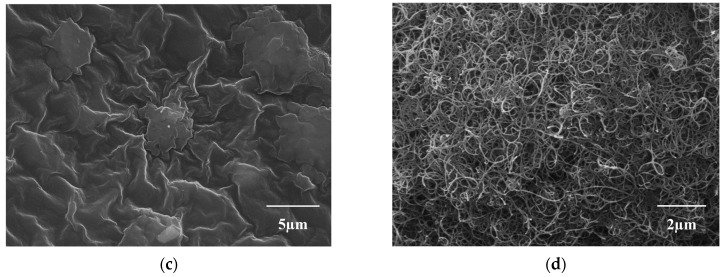
Electrical properties of smart skin: (**a**) conductivity; (**b**) SEM of smart skin (5 wt%); (**c**) SEM of pure PU; and (**d**) SEM of CNTs (lengths: 5–20 microns).

Figure 3a shows the electrical conductivity of the smart skin as a function of the CNT concentration. The electrical conductivity of pure PU is approximately 1 × $10^{-10}$ S/m, indicating its insulating characteristics. As the conductive nanomaterial CNT was reinforced within the PU matrix, a percolation threshold was observed near 1 wt% CNT. An increase in electrical conductivity from 1 wt% to 5 wt% CNT was observed, indicating the formation of conductive pathways within the PU matrix as the CNT concentration increased [34,35,36]. Beyond 5 wt% CNT, even if the CNT concentration was increased, the conductive path became saturated, and there was no significant change in electrical conductivity. This behavior suggests that the conductive pathways among the CNTs within the PU matrix became saturated, leading to minimal changes in conductivity with further increases in CNT concentration. Figure 3b shows the scanning electron microscope (SEM) image of smart skin, showcasing the well-established conductive pathways among the CNTs embedded in the PU matrix. Additionally, to aid understanding, SEM images of the pure PU matrix and CNTs were indeed acquired as negative controls. Specifically, Figure 3c shows the SEM image of the pure PU matrix, while Figure 3d presents the SEM image of the CNTs.

### 3.2. Electro-Mechanical Characterization of Smart Skin

**Figure 4:**
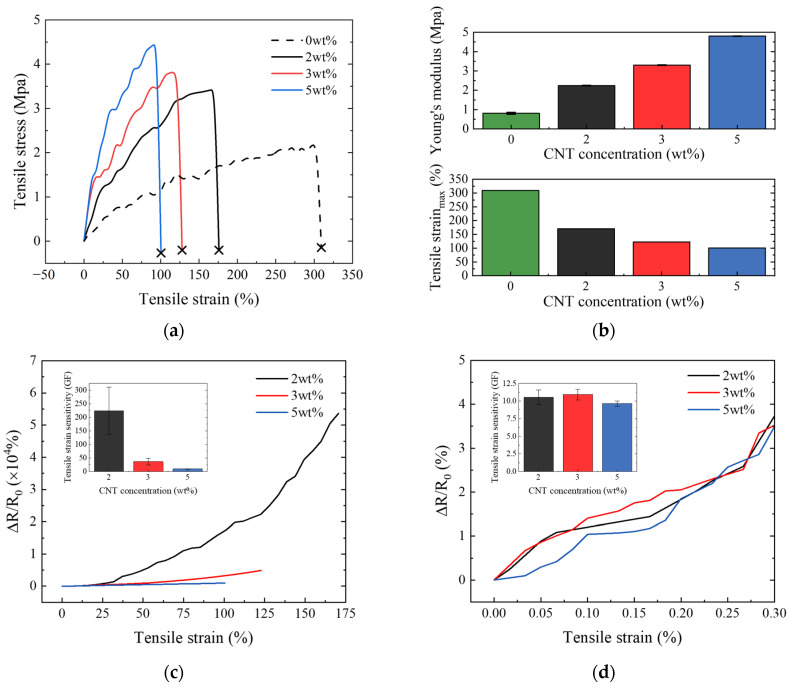
(**a**) Stress–strain curve; (**b**) maximum tensile strain and Young’s modulus; (**c**) fractional change in electrical resistance at full-range strain; and (**d**) fractional change in electrical resistance at fracture strain.

Compared to pure PU, adding 5 wt% CNT significantly improved performance, increasing the maximum tensile stress from 2.1% to 4.5%, representing an approximate enhancement of 114%. Figure 4b demonstrates that the addition of CNTs led to an increase in Young’s modulus and a decrease in ultimate tensile strain. The maximum tensile strain reduced from 309% to 101%, a decline of about 67%. This tendency is primarily attributable to the inherent high strength and stiffness of CNTs. When CNTs are added to a PU matrix, a more robust composite material is produced. Additionally, the smart skin, fabricated using an ultrasonicator, ensures the even dispersion of CNTs within the PU matrix, thereby enhancing interactions such as interfacial adhesion between PU and CNTs [37,38,39,40,41,42]. Importantly, with respect to the research goal of detecting cracks in RC structures, even though the tensile strain of the smart skin was reduced, it achieved this goal by having an ultimate tensile strain that was much higher than the critical failure strain of concrete, which is 0.3%.

Figure 4c,d show the electrical behavior of the smart skin across the full-range strain and at the fracture strain, respectively. It can be clearly seen that the electrical resistance of all samples increased with increasing tensile loading because of the loss of contact between CNTs and the widening of the inter-CNT distances [43,44,45]. At full-range strain, a rapid increase in electrical behavior in electrical resistance was observed as the concentration of CNT increased. At the fracture strain, all samples exhibited a similar behavior in electrical resistance. The tensile strain sensitivity of the smart skin was calculated by dividing the fractional change in electrical resistance by the tensile strain and is shown in the small graphs of Figure 4c,d. At full-range strain, lower concentrations of CNT correspond to significantly higher values of tensile strain sensitivity. At fracture strain, the tensile strain sensitivity of all samples consistently remained around 10. This is because at lower CNT concentrations, the conductive pathways were less stable and more susceptible to disruption or alteration due to strain. As shown in Table 1, all samples of the smart skin exhibited a strain sensitivity more than 3.1 times higher than that of conventional metal-type strain gauges [46,47].

### 3.3. Electro-Environmental Characterization of Smart Skin

**Figure 5:**
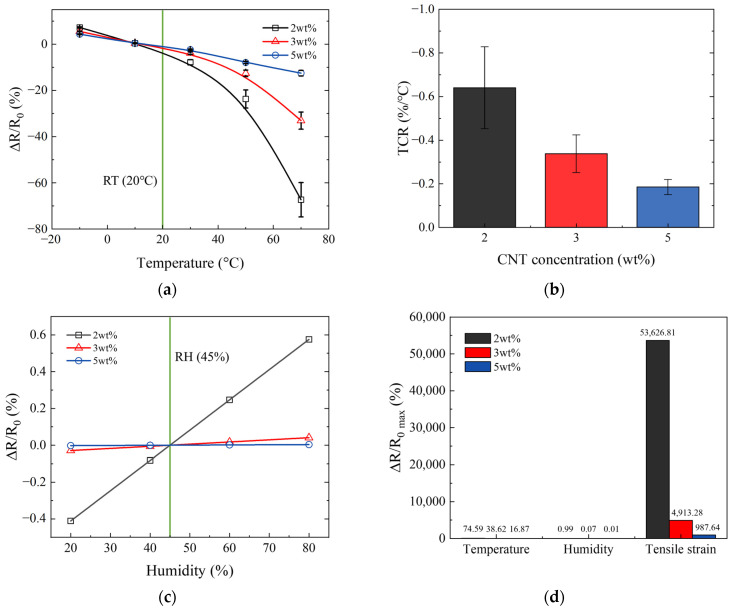
Electro-environmental characterization of smart skin: (**a**) temperature vs. change in electrical resistance (green line: RT 20 °C); (**b**) TCR vs. CNT concentration; (**c**) humidity vs. change in electrical resistance (green line: RH 45%); and (**d**) comparison of the maximum value of change in electrical resistance due to temperature, humidity, and tensile strain.

The smart skin applied to the surface of structures can be exposed to a wide range of environmental conditions. This exposure necessitates a thorough investigation into how various environmental factors affect the electrical behavior of the smart skin. Figure 5 reports the relative change in the electrical resistance of the smart skin due to temperature and humidity, and ultimately compares it with the relative change in electrical resistance caused by tensile strain.

Figure 5a shows the electrical properties of the smart skin with respect to temperature changes. The temperature was adjusted within the range of −10 °C to 70 °C, starting from a baseline of 20 °C, considered room temperature (RT), highlighted by a green line in the graph. All samples exhibited behavior where electrical resistance decreased as the temperature increased. The higher the CNT concentration in the smart skin, the smaller the relative change in electrical resistance due to temperature changes. These findings have been summarized and represented as a negative temperature coefficient of resistance (TCR) in Figure 5b. This behavior occurs because an increase in temperature leads to an increase in electron activity, which consequently reduces the electrical resistance of the smart skin [48,49].

Figure 5c shows the electrical properties of the smart skin in response to changes in humidity. The humidity was adjusted within the range of 20% to 80%, starting from a baseline of 45%, considered room humidity (RH), highlighted by a green line in the graph. All samples showed behavior where electrical resistance increased as the humidity increased. This increase occurs because the composite expands as the humidity rises. Overall, the higher the CNT concentration, the lower the environmental sensitivity of the smart skin was observed. This is because a higher CNT concentration leads to an increase in the conductive network within the composite and a higher stiffness of the composite, resulting in minimal changes in the spacing between CNTs due to environmental changes [50,51,52,53].

Figure 5d presents the results comparing the maximum rate of change in electrical resistance due to environmental changes and tensile strain. The smart skin showed a significantly higher change in electrical resistance due to tensile strain compared to environmental changes. This suggests that even if environmental changes occur, if tensile strain is applied, the changes in electrical resistance due to environmental factors can be disregarded.

### 3.4. Application of Smart Skin to RC Structure

**Figure 6:**
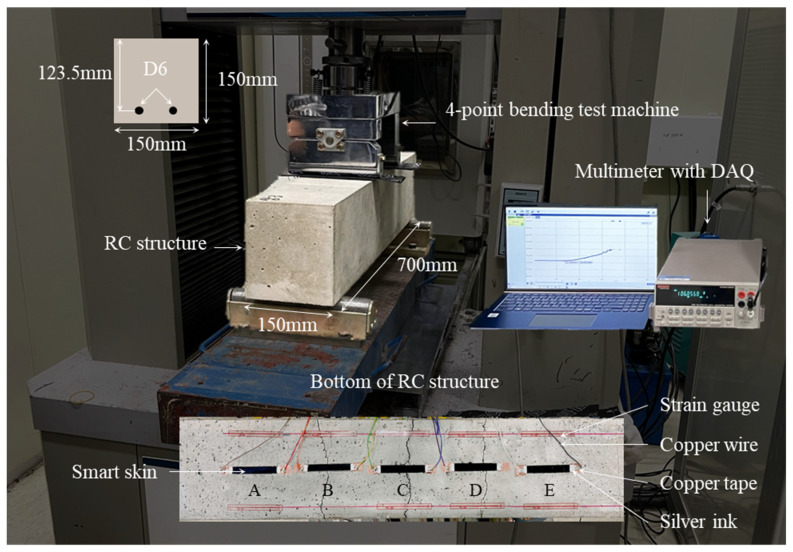
Test setup of the smart skin applied to an RC structure.

Figure 6 shows the test setup of the smart skin applied to an RC structure. To validate the functionality of the smart skin as a crack sensor, a four-point bending test was performed on a rectangular RC structure measuring 700 mm in length, 150 mm in width, and 150 mm in height. The test was conducted at a constant displacement rate of 1.2 kN/min until the point of fracture of the RC structure. To check for sections with cracks, the smart skin was coated over five sections on the bottom surface of the RC structure, each section sized at 70 mm in length, 10 mm in width, and 0.7 mm in thickness. The CNT concentration of the smart skin was selected as 5 wt% CNT, which demonstrates stable electrical behavior in response to mechanical deformation and environmental changes. Electrodes were formed at both ends of each section using copper tape, copper wire, and silver ink, and were connected to a Keithley 2700 with DAQ to record the electrical resistance of the smart skin at one-second intervals. Simultaneously, strain gauges were attached near the smart skin to measure the width of cracks developed in the RC structure.

**Figure 7:**
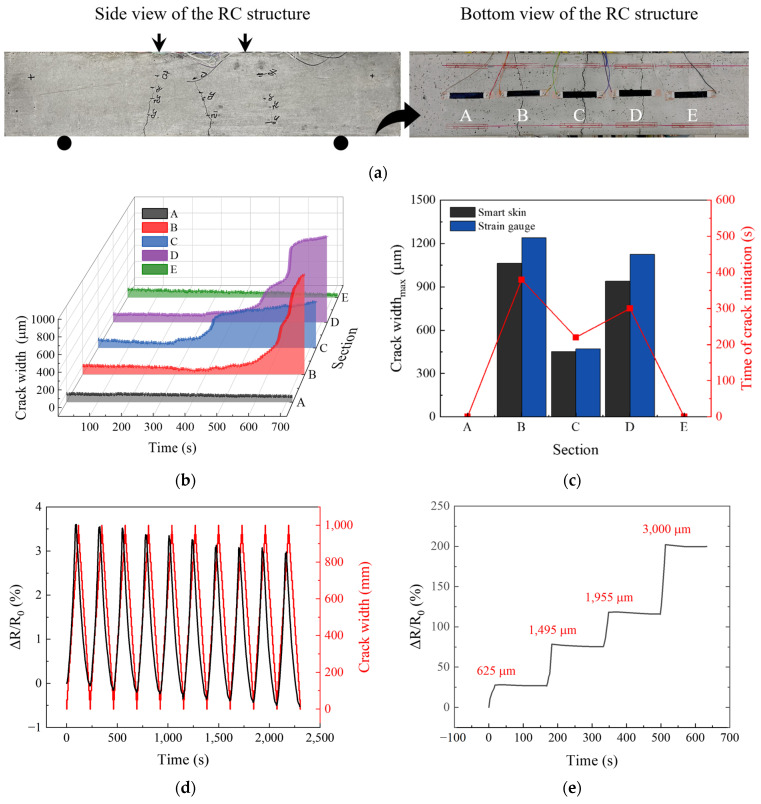
(**a**) Image depicting the occurrence of cracks by section in the RC structure after testing (left image: side view of the RC structure, right image: bottom view of the RC structure, arrows indicate the direction of applied load); (**b**) change in crack width over time by section; (**c**) comparison between smart skin and strain gauge measurements; (**d**) electrical resistance oscillation across repeated crack cycles; and (**e**) electrical response of smart skin to various crack widths during stepwise testing (red text: the size of cracks at the peak of electrical resistance changes).

Figure 7a shows the image of the RC structure after the test, with cracks occurring. Cracks were observed in sections B, C, and D, with the largest crack width found in section B. After the growth of the cracks, the smart skin in all sections remained intact without any damage. Particularly in section B, the strain gauge failed to withstand the crack growth and detached, in stark contrast to the performance of the smart skin.

The crack width for each section over time was derived from the electrical resistance values of the smart skin, which were measured at one-second intervals, and from the tensile strain sensitivity determined in 3.2, as shown in Figure 7b. The comparison of the measurements recorded by the smart skin and the strain gauge is presented in Figure 7c. The cracks occurred in the order of sections C, D, and B, and both the smart skin and the strain gauge indicated the same time of crack initiation. The comparison of the maximum crack widths measured by the smart skin closely matched those recorded by the strain gauges in each section. Furthermore, regression analysis was conducted to compare the crack data measured by the smart skin with those measured by strain gauges, and the Pearson correlation coefficient value was derived, as presented in Table 2. The interpretation of the correlation coefficient values is further elucidated in Table 3 [54,55,56]. Across all sections, the correlation coefficient values exceeded 0.90, indicating a very high correlation. The reason for not achieving a value closer to 1.00 is that the strain gauges were not attached directly next to the smart skin, leading to potential discrepancies in the sizes of the cracks detected by the smart skin and the strain gauges. The smart skin failed to detect bending deformations that occurred before the onset of cracks because it had low sensitivity due to the 5 wt% CNT used in this test. This result demonstrates the potential of the smart skin to monitor only the occurrence and growth of cracks in structures, without detecting bending deformations of the structures.

Additionally, as crack detection sensors need to be able to accommodate the growth of complex cracks, reproducibility becomes a crucial factor to consider in their design. Additional analysis was performed over 10 cycles within the crack range of 0 μm to 1000 μm, resulting from the results in Figure 7b, as shown in Figure 7d. The results indicated that the electrical resistance of the smart skin oscillated between −1.3% and 12.0%. The minimum value averaged −0.7% and the maximum value averaged 11.0%, demonstrating good stability. Furthermore, the smart skin should exhibit good signal stability under different crack widths to ensure reliable performance in complex working conditions. Figure 7e presents the results of stepwise tests at various crack widths. The smart skin showed electrical changes only during the crack formation stage and no electrical changes were observed when the cracks did not grow. This emphasizes the signal stability and reliability of the smart skin as a crack detection sensor and presents its potential applicability as an SHM sensor.

## 4. Conclusions

The results of this study confirm that smart skin is promising as a crack detection sensor for RC structures in civil engineering applications. The concentration of CNT is a key factor that can influence the various properties of the smart skin. Through electro-mechanical/environmental characterization tests, the electrical behavior of the smart skin at different CNT concentrations was analyzed. Overall, a CNT concentration of 5 wt% was determined to be suitable for crack detection sensors in RC structures. This suitability is attributed to its appropriate tensile strain sensitivity and its stable electrical resistance behavior under mechanical deformation and environmental changes. Based on these findings, the smart skin was applied to RC structures and effectively monitored the formation and growth of cracks. It demonstrated excellent reproducibility during cycles of crack width changes, and its reliability as a crack detection sensor was further confirmed through electrical stability throughout these cycles. The presented results validate the smart skin as a crack detection sensor for actual RC structures and demonstrate its potential application as part of an SHM system suitable for integration with IoT systems and implementation in smart cities. Future research will evaluate the material’s resistance to thermal and ultraviolet degradation when applied on concrete surfaces exposed to varying seasonal temperatures and solar intensities. This direction aims to ascertain the smart skin’s maintenance of critical properties under such conditions, ensuring its long-term efficacy and reliability.

## Figures and Tables

**Table 1:** Tensile strain sensitivity vs. CNT concentration.

CNT Concentration (wt%)	Full-Range Strain		Fracture Strain		Metal-Type Strain Gauge Sensitivity
	Sensitivity	Error Range	Sensitivity	Error Range	
2	224.0	±87.0	10.5	±1.0	2.0–3.2
3	36.5	±11.8	10.9	±0.8	
5	9.8	±0.9	9.6	±0.4	

**Table 2:** Section-wise Pearson correlation coefficient values.

Section	Correlation Coefficient (*r*)
A	0.90
B	0.93
C	0.99
D	0.94
E	0.90

**Table 3:** Meaning of Pearson correlation coefficient value.

Scale of Correlation Coefficient (*r*)	Value
0 < *r* ≤ 0.19	Very low correlation
0.20 < *r* ≤ 0.39	Low correlation
0.40 < *r* ≤ 0.59	Moderate correlation
0.60 < *r* ≤ 0.79	High correlation
0.80 < *r* ≤ 1.00	Very high correlation

## Data Availability

Data will be made available on request.
